# Prenatal and childhood exposure to chlordecone and adiposity of seven-year-old children in the Timoun mother–child cohort study in Guadeloupe (French West Indies)

**DOI:** 10.1186/s12940-022-00850-2

**Published:** 2022-04-19

**Authors:** Nathalie Costet, Antoine Lafontaine, Florence Rouget, Léah Michineau, Christine Monfort, Jean-Pierre Thomé, Philippe Kadhel, Luc Multigner, Sylvaine Cordier

**Affiliations:** 1 Univ Rennes, Inserm, EHESP, Irset (Institut de Recherche en Santé, environnement et travail) -UMR_S 1085, F-35000 Rennes, France; 2 CHU de Rennes, Univ Rennes, Inserm, EHESP, Irset (Institut de Recherche en Santé, environnement et travail) - UMR_S 1085, F-35000 Rennes, France; 3 Univ Rennes, Inserm, EHESP, Irset (Institut de Recherche en Santé, environnement et travail) -UMR_S 1085, F-97100 Pointe-à-Pitre, France; 4 LEAE-CART (Laboratoire d’Ecologie Animale Et d’Ecotoxicologie-Centre de Recherche Analytique Et Technologique), Université de Liège, Liège, Belgium; 5 CHU de Guadeloupe, Univ Antilles, Inserm, EHESP, Irset (Institut de Recherche en Santé, environnement et travail) - UMR_S 1085, F-97100 Pointe-à-Pitre, France

**Keywords:** Adiposity, Obesity, Chlordecone, Organochlorine pesticides, Prenatal exposure, Childhood exposure, Biomonitoring

## Abstract

**Background:**

Exposure to persistent environmental organic pollutants may contribute to the development of obesity among children. Chlordecone is a persistent organochlorine insecticide with estrogenic properties that was used in the French West Indies (1973–1993) and is still present in the soil and the water and food consumed by the local population. We studied the association between prenatal and childhood exposure to chlordecone and the adiposity of prepubertal children.

**Methods:**

Within the Timoun Mother–Child Cohort Study in Guadeloupe (French West Indies), 575 children had a medical examination at seven years of age, including adiposity measurements. A Structural Equation Modeling approach was used to create a global adiposity score from four adiposity indicators: the BMI z-score, percentage of fat mass, sum of the tricipital and subscapular skinfold thickness, and waist-to-height ratio. Chlordecone concentrations were measured in cord blood at birth and in the children’s blood at seven years of age. Models were adjusted for prenatal and postnatal covariates. Sensitivity analyses accounted for co-exposure to PCB-153 and pp’-DDE. Mediation analyses, including intermediate birth outcomes, were conducted.

**Results:**

Prenatal chlordecone exposure tended to be associated with increased adiposity at seven years of age, particularly in boys. However, statistical significance was only reached in the third quartile of exposure and neither linear nor non-linear trends could be formally identified. Consideration of preterm birth or birth weight in mediation analyses did not modify the results, as adjustment for PCB-153 and pp’-DDE co-exposures.

**Conclusion:**

Globally, we found little evidence of an association between chlordecone exposure during the critical in utero or childhood periods of development and altered body-weight homeostasis in childhood. Nevertheless, some associations we observed at seven years of age, although non-significant, were consistent with those observed at earlier ages and would be worth investing during further follow-ups of children of the Timoun Mother–Child Cohort Study when they reach puberty.

**Supplementary Information:**

The online version contains supplementary material available at 10.1186/s12940-022-00850-2.

## Background

The prevalence of obesity has been rising over the last few decades and is now a major public health issue among children and adults [1]. Childhood obesity is of concern because it is a significant driver of adverse health effects at a later age. These include diabetes mellitus, cardiovascular diseases, hypertension and stroke, and certain types of cancer [2, 3]. Obesity results from a combination of factors, including genetics, diet, and physical activity. Experimental data have suggested that perinatal exposure to certain environmental chemical contaminants can interfere with energy balance, encouraging weight gain and early-onset obesity [4, 5]. Particular attention has focused on substances with hormonal properties (endocrine-disrupting chemicals, EDCs) because of their ability to disrupt hormonally-regulated metabolic processes, especially if exposure occurs during early development [6]. Among them, persistent organic pollutants (POPs) have attracted attention because of their widespread presence in humans due to their high persistence and low environmental degradability.

Several epidemiological studies have investigated relationships between early exposure to POPs (in utero and during childhood) and the risk of childhood obesity. Most used a single indicator, such as the body-mass index (BMI) or weight [7–10]. Fewer studies considered other indicators of adiposity, such as waist circumference, waist-to-height ratio, or skinfold thickness [11, 12]. Consistent associations have been reported between prenatal exposure to p,p´‑dichlorodiphenyldichloroethylene (DDE, the major and most persistent metabolite of dichlorodiphenyltrichloroethane, DDT) and an elevated BMI in childhood, whereas associations with prenatal polychlorinated biphenyl (PCB) exposure has been less consistently reported [13–17].

Chlordecone (decachloroocta-hydro-1,3,4,-metheno-2H-cyclobuta[cd]-pentalene-2-one, also known as Kepone) is an organochlorine insecticide initially manufactured in the United States in the early 1960s [18]. After the chemical disaster that resulted in a poisoning episode involving chlordecone plant workers in the industrial city of Hopewell (Virginia) [19] and the contamination of the James River up to the Chesapeake Bay, chlordecone use, production, and distribution was banned by the United States in 1976 [20]. However, it was subsequently produced by a French company and extensively used to control the banana root borer from 1973 to 1993 in the French West Indies (FWI, Guadeloupe and Martinique) [21]. This pesticide undergoes no significant biotic or abiotic degradation in the environment^18^, and it has been estimated that the duration of chlordecone pollution of soil in FWI will last for decades or centuries [22]. Chlordecone in soil is slowly drained by rainfall towards superficial water, ground water, and marine coastal waters and contaminates the terrestrial and aquatic ecosystems, including crops, livestock, and fishing products [23, 24]. The permanently polluted soil and water are thus responsible for the contamination of local foodstuffs and the population of FWI, including pregnant women and children, continues to be exposed to this chemical through the consumption of contaminated foodstuffs [25].

Numerous toxicological studies have demonstrated that chlordecone is a reproductive and developmental toxicant and neurotoxic and carcinogenic in rodents [18]. It is also an EDC because of its estrogenic properties, both in vitro and in vivo [26, 27]. Chlordecone crosses the placental barrier in pregnant rodents and is transferred to the newborn through maternal breastfeeding, thus exposing the developing organism during the earliest stages of development [28]. Moreover, early post-natal exposure to chlordecone in rats is associated with significant sex-dependent changes in adult body weight, resulting in lighter males and heavier females [29].

The Timoun Mother–Child Cohort Study was established in Guadeloupe (FWI) to investigate the consequences of prenatal and childhood exposure to chlordecone on pregnancy and child development. In previous analyses of the Timoun Mother–Child Cohort Study, we showed that prenatal exposure of children to chlordecone, assessed by chlordecone concentrations in the cord blood, may be associated with reduced birth weight in overweight and obese mothers in a non-monotonic manner [30]. We further observed that prenatal exposure to chlordecone was associated with an elevated BMI in early infancy (in boys at 3 months of age and in girls at 8 and 18 months of age), whereas early postnatal exposure to chlordecone via breastfeeding and contaminated foodstuffs was associated with lower BMI measurements at 3, 8, and 18 months, especially in girls [31].

Here, we investigated whether the association between prenatal and childhood exposure to chlordecone and adiposity was still present at a later age, among seven-year-old children, in the same Timoun Mother–Child Cohort Study. We used a multidimensional approach to estimate adiposity by combining four indicators: BMI z-score, the sum of the skinfolds, the weight-to-height ratio (WHtR), and the percentage of body fat mass.

## Methods

### Population

**Fig. 1:**
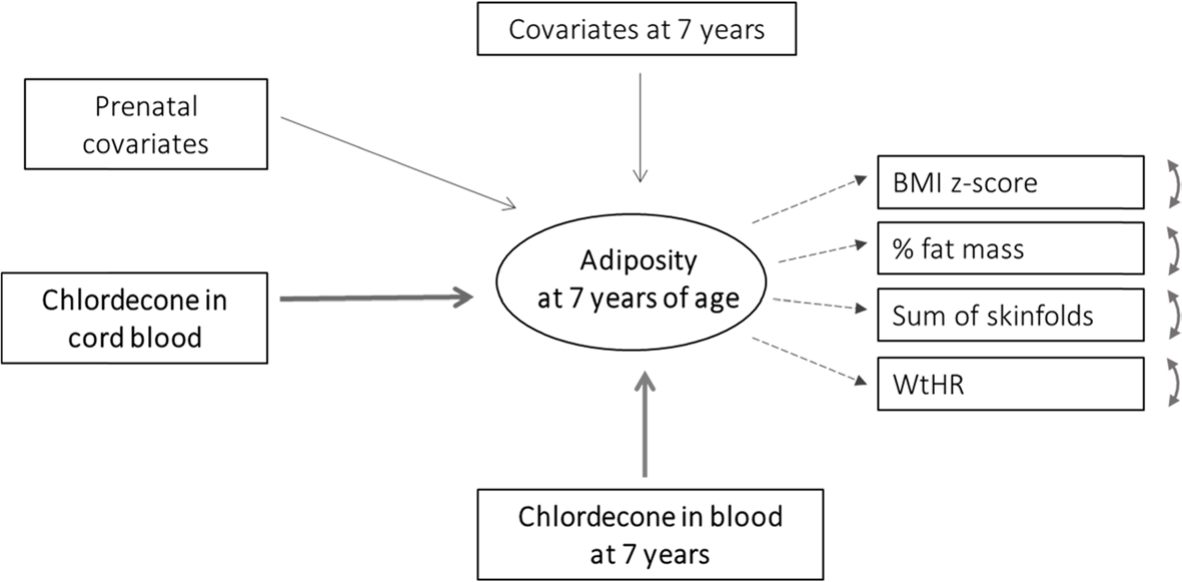
Graphical representation of the Structural Equation Modeling of the association between exposure to chlordecone and adiposity at age 7. Note: Adiposity is a latent trait defined by four indicators. Adiposity indicators: BMI z-score: WHO references, by sex and age (in months); % Fat mass: estimated by bioelectrical impedance; Sum of skinfolds: sum of the subscapular and tricipital fold measurements; WHtR (Waist-to-Height Ratio): waist circumference (cm) / height (cm). Arrows with solid lines represent regression coefficients. Arrows with dotted lines represent factor loadings. Double-headed curvilinear arrows represent residual variances of the indicators. Prenatal covariates: maternal place of birth, maternal BMI before pregnancy, and maternal level of education, cord blood total lipids (g/L, log_10_ scale). Covariates at 7 years of age: exact age at measurement, duration of breastfeeding, time spent exercising, watching TV, or playing videogames per week, and obesogenic dietary habits

This study took place in Guadeloupe (FWI), a Caribbean archipelago where most of the inhabitants are of African descent. The Timoun Mother Child Cohort Study included 1,068 pregnant women between November 2004 and December 2007 from the general population, recruited during last-trimester visits at public and private health centers (University Hospital of Guadeloupe, General Hospital of Basse-Terre, Polyclinic of Guadeloupe, and antenatal care dispensaries) [32]. At inclusion, women were interviewed by trained midwives to assess their medical history, socioeconomic conditions, and dietary habits. At delivery, data concerning maternal diseases during pregnancy, the delivery, and newborn health status and anthropometric characteristics at birth were collected by the medical staff and maternal and cord blood samples were obtained. Follow-up visits of the children were then organized at 3, 7, and 18 months of age in a selected sub-cohort excluding cases of multiple birth, preterm birth, intra-uterine growth restriction, neonatal disease or malformation, and serious maternal illness before or during pregnancy (*N* = 611) [33–35]. At seven years of age (May 2011-October 2015), all mothers initially included in the cohort were contacted for a follow-up interview and a medical examination of their child. Among them, 592 (55.4% of the initial cohort) were interviewed and had their child examined at the University Hospital of Guadeloupe. During the medical examination of the children, face-to-face interviews were conducted with their mothers to collect information about the socio-economic context in which the child was growing up (environment, lifestyle, dietary habits, etc.) and the health of the child and his/her parents. A blood sample was also collected from the children at the end of the examination. For the present study, we excluded 17 children because of major congenital anomalies or severe diseases that may affect growth (*N* = 4) and those with initiated puberty (breast Tanner stage ≥ 3 for girls and testis volume ≥ 5 ml for boys) (*N* = 13), leaving 575 children in the final sample (S-Fig. 1).

### Exposure assessment

Cord blood and child blood samples at the seven-year visit were collected in EDTA tubes. After centrifugation, plasma samples were stored at -20 °C. They were transferred by airmail on dry ice to Liège, Belgium, for organochlorine analysis. Chlordecone, p,p’-DDE, and PCB congener 153 (PCB-153) analyses were performed by the Center for Analytical Research and Technology (CART) at Liège University in Belgium, in 2007–2009 or 2013 for cord blood samples and in 2015 for child blood samples.

Among PCBs, we selected congener 153 because it correlates very well with the total PCB concentration in plasma [36]. The concentrations in plasma were quantified by gas chromatography coupled to electron-capture detection, as previously described [37, 38]. The limit of detection (LOD) was 0.06 μg/L for cord chlordecone, 0.05 µg/L for cord p,p’-DDE, and PCB-153, and 0.02 μg/L for childhood chlordecone, p,p’-DDE, and PCB-153.

Total cord plasma cholesterol and triglyceride concentrations were determined enzymatically (DiaSys Diagnostic Systems GmbH; Holzheim, Germany) and the total lipid concentration calculated as previously described [39].

### Adiposity indicators at seven years of age

During the medical examination, anthropometric measurements were taken by trained nurses or midwives*,* following a standardized protocol, to assess the child’s growth, corpulence, and fat repartition. They included height, waist, and hip circumference measurements, and tricipital and subscapular skinfold thickness. Each measurement was performed twice and averaged for the analyses. The weight and body fat mass were estimated once by bioelectrical impedance using a professional body composition monitor (Tanita® BC.420.MA-S scale, Tokyo). We considered four indicators to assess adiposity of the children for the present study: (1) the BMI, computed as the ratio of the weight (kg) to squared height (m^2^), which was transformed into a z-score according to the sex and exact age of the child at the time of the measurement using the WHO references, [40] (2) the waist circumference, measured while the child was standing upright, in exhalation, halfway between the last rib and the iliac crest, from which the WHtR was computed, (3) the sum of skin folds (mm), calculated from the subscapular and tricipital fold measurements, and (4) the body fat mass, expressed as the percentage of fat mass (%).

### Statistical analysis

We used the structural equation modeling (SEM) framework to study the association between prenatal and childhood exposure to chlordecone and the children’s adiposity at seven years of age, estimated through multiple indicators. The “adiposity” latent variable (called the “adiposity score” throughout the manuscript) was based on the BMI z-score, the sum of the skinfolds, the WHtR, and the percentage of body fat mass. The percentage of fat mass and sum of the skinfolds were log_10_-transformed to normalize their distribution. For easier interpretation of the adiposity latent variable, the factor loading of the BMI z-score was arbitrarily set to 1, such that the adiposity score was expressed using the same unit as the BMI z-score. The associations between cord and child blood chlordecone concentrations and the adiposity score were estimated by linear regression coefficients (Fig. 1). All model parameters (factor loadings, variances, covariances, and regression coefficients) were estimated by maximum likelihood. Parameters were considered to be significant if their 95% confidence intervals (95% CIs) did not include 0. As recommended in the SEM approach, a combination of various model fit indices was checked to validate the models [41–43]: a Satorra–Bentler scaled Chi-square statistic with *p* > 0.05, a Root Mean Square Error of Approximation (RMSEA) < 0.06, a Comparative Fit Index (CFI) > 0.90, a Goodness-of-Fit statistic (GFI) > 0.95, and a Standardized Root Mean Square Residual (SRMR) < 0.05 were considered to establish a good fit of the model.

Cord and child blood chlordecone concentrations were considered as categorical (quartiles, based on their distribution in the population study) or continuous variables for the analyses. As continuous variables, chlordecone values under the LOD were replaced by random values below the LOD using the maximum likelihood method [44, 45]. The imputation of values below the LOD was run 10 times so that 10 imputed samples were produced and analyzed after log_10_-transformation of cord blood and childhood concentrations.

The a priori selection of covariates used for adjustment in the models was based on those found in the literature on the association of perinatal exposure to POPs with child growth and the risk of childhood obesity [46–49]: exact age at measurement (in months), maternal place of birth (Guadeloupe/Martinique, other Caribbean Islands, Europe), maternal BMI before pregnancy (< 18.5, 18.5 to < 25, 25 to < 30, ≥ 30 kg/m^2^), maternal level of education (< 5, 5 to < 12, ≥ 12 years), duration of (non-exclusive) breastfeeding (none, < 3, 3 to < 7, 7 to < 18 months, ≥ 18 months), time spent exercising per week (no activity, ≤ median, > median duration among those practicing a sport), time spent watching TV or playing videogames per week (≤ median, > median), and dietary habits of the child. Information on the child's diet at seven years of age was collected using a frequency food questionnaire (28 foodstuffs, frequencies ranging from "never" to "several times a day"). We defined a score of obesogenic food consumption by summing the frequencies of intake of the following foodstuff categories: ‘French fries, fried potatoes', ‘pizzas, pies', 'soft drinks', 'light soda', ‘pastries, biscuits, cakes', 'sweets, chocolate', and 'crackers, snacks'. The score for obesogenic dietary habits was categorized into quartiles. All models were adjusted for total lipids in cord blood (when studying prenatal exposure) or in child blood (when studying postnatal exposure), after log10-transformation [50, 51]. Missing data for chlordecone concentrations and covariates were accounted for using the Full Information Maximum Likelihood (FIML) method [52, 53].

All analyses were stratified by sex because of morphological differences between boys and girls and a potential interaction with chlordecone exposure.

Sensitivity analyses were conducted: models for prenatal chlordecone exposure were adjusted for cord blood PCB-153 and p,p’-DDE levels and for child chlordecone levels; similarly, models for childhood chlordecone exposure were further adjusted for child PCB-153 and p,p’-DDE levels at the same age and for cord chlordecone level. We also tested the role of intermediate outcomes as mediator variables by conducting formal mediation analyses that included the following birth outcomes that could potentially result in an association between prenatal exposure and adiposity at seven years of age: [54–56] birth weight, preterm birth, small for gestational-age (SGA), and large for gestational-age (LGA). These mediation analyses were performed within the SEM approach to identify the direct and indirect effects of prenatal chlordecone exposure on adiposity at seven years of age.

As complementary analyses, we separately modeled each adiposity indicator using linear regression models to test their consistency with the SEM approach and provide results comparable to those of the literature. We also implemented General Additive Models (GAM), including restricted cubic splines, to fit a potential non-linear association between the multidimensional adiposity score and exposure [57].

We finally checked correlations between covariates (S-Table 1) and verified that there was no multi-collinearity issue when estimating the models (not shown).

The analyses were computed using SAS® and R software. SEM analyses were conducted with the *lavaan* R package [58].

## Results

Most (~ 90%) of the mothers of children participating in the follow-up of the Timoun Mother–Child Cohort Study were of Caribbean origin (French West Indies, other Caribbean islands) (Table 1). Approximately 41% of these women were overweight or obese before pregnancy, 14% had diabetes mellitus (non-gestational or gestational), and 13% had hypertension (non-gestational or gestational). Tobacco smoking and alcohol consumption during pregnancy were reported by only 3 and 2% of the mothers, respectively. Fourteen percent of the children were born preterm, whereas 9% each were considered SGA or LGA. Children were examined at an average age of seven years and eight months. All attended school and 47% practiced a sport outside of school (weekly median practice time = 2 h, when practicing). They spent on average of 11 h/week (including school days and weekends) watching TV and 70% played videogames (median = 3.5 h/week among players).

**Table 1:** Description of the study sample

	**All (*N* = 575)**		**Boys (*N* = 283)**		**Girls (*N* = 292)**		***p*-value**^**a**^
	N	% or mean (std)	N	% or mean (std)	N	% or mean (std)	
**Pregnancy and birth**							
Maternal age	575	31.7 (6.6)	173	31.8 (6.8)	200	31.7 (6.4)	0.59
Maternal place of birth							0.06
French West Indies	465	80.9	238	84.1	227	77.7	
Other Caribbean Islands	52	9.0	25	8.8	27	9.2	
Europe	58	10.1	20	7.1	38	13.0	
Maternal education (yrs)							0.54
< 5	28	4.9	13	4.6	15	5.1	
5–12	398	69.2	202	71.4	196	67.1	
> 12	149	25.9	68	24.0	81	27.7	
Maternal BMI							0.57
Underweight	33	5.8	19	6.8	14	4.9	
Normal	300	53.1	151	54.3	149	51.9	
Overweight	131	23.2	63	22.7	68	23.7	
Obese	101	17.9	45	16.2	56	19.5	
Tobacco during pregnancy	18	3.1	8	2.8	10	3.4	0.68
Alcohol during pregnancy	12	2.2	5	1.9	7	2.5	0.61
Hypertension^**b**^	70	12.6	30	10.9	40	14.2	0.25
Diabetes^**c**^	76	13.7	41	15.0	35	12.5	0.39
Preterm birth	83	14.4	45	15.9	38	13.0	0.32
Small for gestational age	51	8.9	31	10.9	20	6.8	0.08
Large for gestational age	51	8.9	18	6.4	33	11.3	0.04
**Child at 7 years of age**							
Age at examination (months)	575	92.0 (2.6)	283	92.1 (2.7)	292	91.9 (2.6)	0.54
BMI z-score							0.001
Underweight	17	3.0	12	4.3	5	1.7	
Normal	406	70.9	195	69.2	211	72.5	
Overweight	87	15.2	33	11.7	54	18.6	
Obese	63	11.0	42	14.9	21	7.2	
% Fat mass	565	19.4 (6.2)	276	18.2 (5.8)	289	20.4 (6.5)	< 0.001
Subscapular fold (mm)	495	6.8 (3.1)	240	6.3 (2.8)	255	7.3 (3.3)	< 0.001
Tricipital fold (mm)	510	9.6 (4.1)	249	8.6 (3.9)	261	10.5 (4.1)	< 0.001
Sum of skinfolds (mm)	495	16.5 (6.8)	240	15.0 (6.4)	255	17.9 (6.9)	< 0.001
Waist circumference (cm)	571	57.4 (6.8)	283	57.4 (6.8)	288	57.4 (6.9)	0.80
Waist-to-height ratio	571	0.44 (0.04)	283	0.44 (0.04)	288	0.44 (0.05)	0.63
Sport practice (Yes)	271	47.3	140	49.8	131	44.9	0.23
Time practicing sports (h/week) ^d^	271	2 (1.5 – 4)	140	2 (2.5 – 4)	131	2 (1 – 3.5)	0.03
Time watching TV (h/week)	575	11.4 (7.5)	283	11.1 (7.2)	292	11.8 (7.7)	0.34
Time playing videogames (h/week)	575	3.2 (4.0)	283	3.5 (4.0)	292	2.9 (4.0)	0.02

^**a**^*p*-values from Wilcoxon (continuous variables) or Chi-square (nominal variables) tests

^**b**^Non-gestational or gestational hypertension

^**c**^Non-gestational or gestational diabetes

^**d**^Median (Q1- Q3) time of sport practice among practicing children

Among the 575 children, 87 (15%) were overweight and 63 (11%) were obese (Table 1). The distribution of BMI categories varied significantly between boys and girls: girls were significantly more often overweight (19% vs 12% of the boys), but boys were more often obese (15% vs 7% of the girls). The mean percentage of fat mass was 19.4% and was significantly higher for girls (20.4%) than for boys (18.2%). The average sum of the skinfolds, available for 495 children (86% of the sample), was 16.5 mm and significantly higher for girls (18 mm vs 15 mm in boys). The mean WHtR was 0.44 and did not differ between sexes. There was a strong correlation among the four indicators of adiposity (Spearman *p* > 0.70) for both sexes, which tended to be higher for girls (S-Table 2). Factor loadings of these indicators on the adiposity latent variable are presented in S-Table 3. They were all significant and did not vary between the raw (minimally adjusted for age and maternal place of birth) and fully adjusted models. The fit statistics of the SEM models considering both sexes together were partially unsatisfactory (χ^2^ *p* < 0.01, RMSEA *p* = 0.07). After stratification, all fit indicators were satisfactory for both sexes (S-Table 3). The sex-specific distributions of the adiposity latent variable are plotted in S-Fig. 2.

The distributions of chlordecone, PCB-153, and pp’-DDE concentrations in cord blood and the blood of the children at seven years of age are presented in Table 2. The median concentration of chlordecone was 0.21 μg/L (Interquartile Range (IQR) = [0.07; 0.38]) in the cord blood samples and 0.05 μg/L (IQR = [0.02; 0.11]) in the children’s blood samples at seven years of age. There was no significant correlation between chlordecone concentrations in the cord blood of the children and their blood at seven years of age (Spearman *p* = 0.09, *p* = 0.13). Cord blood concentrations of chlordecone did not correlate significantly with cord blood concentrations of PCB-153 (*ρ* = -0.02, *p* = 0.76) and only mildly with cord blood concentrations of pp’-DDE (*ρ* = 0.16, *p* = 0.002). Finally, blood concentrations of chlordecone at seven years of age mildly correlated (negatively) with blood concentrations of PCB-153 or did not correlate significantly with blood concentrations of p,p’-DDE at the same age (*ρ* = -0.11, *p* = 0.02 and ρ = 0.004, *p* = 0.92, respectively) (S-Table 4).

**Table 2:** Distributions of chlordecone, PCB153, and pp’-DDE concentrations in cord blood and the blood of the children at seven years of age

Concentration in µg/L		N	LOD	% detected	Min	Q1	Median	Q3	Max
In cord blood	Chlordecone	373	0.06	78.6	< LOD	0.07	0.21	0.38	29. 8
	PCB153	372	0.05	53.0	< LOD	< LOD	0.06	0.14	1.75
	pp’-DDE	372	0.05	83.6	< LOD	0.10	0.28	0.73	12.5
At 7 years (blood)	Chlordecone	446	0.02	71.7	< LOD	0.02	0.05	0.11	7.01
	PCB153	446	0.02	83.9	< LOD	0.04	0.07	0.12	1.29
	pp’-DDE	446	0.02	97.1	< LOD	0.09	0.19	0.42	26.4

*LOD* Limit of detection, *Min* Minimum, *Q1* 1^rst^ Quartile, *Q3* 3^rd^ Quartile, *Max* Maximum

Chlordecone cord blood concentration as a continuous variable (log_10_) was not linearly significantly associated with the adiposity score for boys or girls (Table 3), whether adjusting or not for prenatal co-exposure to PCB-153 and pp’-DDE or childhood exposure to chlordecone (β = 0.20, 95% CI, -0.15 − 0.55 for boys and β = 0.16, 95% CI, -0.13 − 0.45 for girls in Model 1). However, boys and girls had higher adiposity scores in all upper quartiles relative to the first quartile but reaching statistical significance in the third quartile only (β = 0.85, 95% CI, 0.22 − 1.49 for boys and β = 0.49, 95% CI, 0.04 − 0.95 for girls) (Table 3, Model 1). This association was maintained across different adjustment strategies: inclusion of cord blood PCB-153 and pp’-DDE concentrations in the models strengthened the association observed in the second and third quartiles of exposure for boys (β = 0.66, 95% CI, 0.05 − 1.27 for Q2 and β = 0.99, 95% CI, 0.38 − 1.61 for Q3) (Table 3, Model 2). Further adjustment for blood chlordecone concentrations at seven years of age decreased the association observed for the cord blood concentrations among boys and reinforced the association among girls (Table 3, Model 3). Linear regression models separately run for each individual adiposity indicator yielded results consistent with those observed with the SEM approach of adiposity (no significant linear association, increased adiposity, particularly in the third quartile relative to the first quartile, with higher effect-sizes for boys) (S-Table 5).

**Table 3:** Association between chlordecone concentrations in cord blood and adiposity at seven years of age

		**Model 1**^b^	**Model 2**^c^	**Model 3**^d^
**Chlordecone in cord blood (µg/L)**	N	β (95% CI)	β (95% CI)	β (95% CI)
**Boys**	173/283^a^			
< 0.075	40	0 (ref)	0 (ref)	0 (ref)
0.075—0.212	45	0.61 (-0.03; 1.24)	0.66 (0.05; 1.27)	0.47 (-0.20; 1.15)
0.212—0.382	45	0.85 (0.22; 1.49)	0.99 (0.38; 1.61)	0.65 (-0.01; 1.31)
≥ 0.382	43	0.26 (-0.36; 0.88)	0.35 (-0.25; 0.95)	0.29 (-0.37; 0.94)
log_10_	173	0.20 (-0.15; 0.55)	0.17 (-0.18; 0.52)	0.21 (-0.15; 0.57)
**Girls**	200/292^a^			
< 0.075	54	0 (ref)	0 (ref)	0 (ref)
0.075—0.212	49	0.08 (-0.38; 0.53)	0.16 (-0.29; 0.61)	0.20 (-0.30; 0.69)
0.212—0.382	48	0.49 (0.04; 0.95)	0.48 (0.03; 0.92)	0.65 (0.17; 1.13)
≥ 0.382	49	0.31 (-0.16; 0.79)	0.15 (-0.33; 0.63)	0.49 (-0.01; 0.99)
log_10_	200	0.16 (-0.13; 0.45)	0.15 (-0.14; 0.44)	0.16 (-0.13; 0.45)

Adiposity is a latent variable defined from the BMI z-score, % Fat Mass, Sum of subscapular and tricipital folds, and Waist-to-Height Ratio, expressed in the same units as the BMI z-scores

Indices of fit (Model 1, exposure in quartiles):

Boys: χ^2^ *p* = 0.02; RMSEA = 0.04 95% CI, 0.01- 0.05; CFI = 0.98; GFI = 1; SRMR = 0.01

Girls: χ^2^ *p* = 0.21; RMSEA = 0.02 95% CI, 0.00–0.04; CFI = 0.99; GFI = 1; SRMR = 0.01

^a^Missing cord blood concentrations accounted for by the FIML estimation

^b^Model 1 was adjusted for: exact age at measurement (months), maternal place of birth (Guadeloupe / Martinique, other Caribbean Islands, Europe), maternal BMI before pregnancy (< 18.5, 18.5 to < 25, 25 to < 30, ≥ 30 kg/m^2^), maternal level of education (< 5, 5 to < 12, ≥ 12 years), duration of breastfeeding (four categories), time spent exercising per week, time spent watching TV or playing videogames per week, obesogenic dietary habits at seven years of age (four categories), cord blood total lipids (g/L, log_10_ scale)

^c^Model 2 is the same as Model 1 but additionally adjusted for cord blood concentrations of PCB153 and pp’-DDE

^d^Model 3 is the same as Model 1 but additionally adjusted for blood concentrations of chlordecone at age 7 (*N* = 131 boys and *N* = 158 girls with blood samples available at birth (cord blood) and at 7 years)

Non-linear modeling of the associations between prenatal exposure as a continuous variable and the global adiposity score using restricted cubic splines (GAM) showed no significant trend (neither linear nor non-linear) (S-Fig. 3).

The results of mediation analyses are graphically presented in S-Fig. 4 to S-Fig. 7. There was no significant indirect effect through any of the intermediate birth outcomes tested.

Finally, no significant associations were observed between chlordecone concentrations at seven years of age as a continuous variable (log_10_) and adiposity for boys or girls, regardless of the adjustment model (Table 4) (β = -0.07, 95% CI, -0.42 − 0.28 for boys and β = -0.001, 95% CI, -0.29 − 0.29 for girls in Model 1). In the categorical analysis, adiposity tended to be lower in the two upper quartiles of exposure for boys, but without consistent statistical significance.

**Table 4:** Association between chlordecone concentrations and adiposity at seven years of age

		**Model 1**^b^	**Model 2**^c^	**Model 3**^d^
**Chlordecone in blood at 7 years (µg/L)**	N	β (95% CI)	β (95% CI)	β (95% CI)
**Boys**	221/283^a^			
< 0.017	53	0 (ref)	0 (ref)	0 (ref)
0.017—0.051	51	0.20 (-0.35; 0.74)	0.12 (-0.36; 0.60)	0.16 (-0.39; 0.70)
0.051—0.112	62	-0.22 (-0.73; 0.30)	-0.48 (-0.95; -0.01)	-0.16 (-0.68; 0.36)
≥ 0.112	55	-0.07 (-0.61; 0.46)	-0.10 (-0.58; 0.38)	-0.01 (-0.56; 0.53)
log_10_	221	-0.07 (-0,.42; 0.28)	-0,09 (-0.38; 0.20)	-0.08 (-0.43; 0.27)
**Girls**	225/292^a^			
< 0.017	60	0 (ref)	0 (ref)	0 (ref)
0.017—0.051	59	0.20 (-0.23; 0.62)	0.28 (-0.12; 0.69)	0.32 (-0.13; 0.77)
0.051—0.112	49	0.06 (-0.40; 0.51)	0.01 (-0.41; 0.44)	0.01 (-0.45; 0.47)
≥ 0.112	57	-0.10 (-0.54; 0.33)	-0.12 (-0.54; 0.30)	0.01 (-0.44; 0.46)
log_10_	225	-0.001 (-0.29; 0.29)	-0,07 (-0.36; 0.22)	0.01 (-0.28; 0.30)

Adiposity is a latent variable defined from the BMI z-score, % Fat Mass, Sum of subscapular and tricipital folds, and Waist-to-Height Ratio, expressed in the same units as the BMI z-scores

Indices of fit (Model 1, exposure in quartiles):

Boys: χ^2^ *p* = 0.02; RMSEA = 0.04 95% CI, 0.02–0.05; CFI = 0.98; GFI = 1; SRMR = 0.01

Girls: χ^2^ *p* = 0.06; RMSEA = 0.03 95% CI, 0.00–0.05; CFI = 0.99; GFI = 1; SRMR = 0.01

^a^Missing blood concentrations at seven years of age accounted for by the FIML estimation

^b^Model 1 was adjusted for: exact age at measurement (months), maternal place of birth (Guadeloupe / Martinique, other Caribbean Islands, Europe), maternal BMI before pregnancy (< 18.5, 18.5 to < 25, 25 to < 30, ≥ 30 kg/m^2^), maternal level of education (< 5, 5 to < 12, ≥ 12 years), duration of breastfeeding (four categories), time spent exercising per week, time spent watching TV or playing videogames per week, obesogenic dietary habits at seven years of age (four categories), blood total lipids at 7 years of age (g/L, log_10_ scale)

^c^Model 2 is the same as Model 1 but additionally adjusted for blood concentrations of PCB153 and pp’-DDE

^d^Model 3 is the same as Model 1 but additionally adjusted for cord blood concentrations of chlordecone (*N* = 131 boys and *N* = 158 girls with blood samples available at birth (cord blood) and at 7 years)

## Discussion

In the Timoun Mother–Child Cohort Study, we examined the association between prenatal and postnatal chlordecone exposure and a global adiposity score in school-age children. We found a non-monotonic association between prenatal exposure to chlordecone, assessed by concentrations in cord blood, and adiposity at seven years of age for both boys and girls. Only the third quartile of prenatal exposure was associated with significantly increased adiposity, more markedly in boys. Further adjustments for PCB-153 and DDE did not change substantially the results. The association was not mediated by the birth weight of the child or his/her status at birth, such as being preterm, SGA, or LGA. The association between postnatal exposure to chlordecone, assessed by concentrations in the children’s blood at seven years of age, and adiposity at the same age tended to be negative among boys, but non-significantly.

The age of the children in the present study (seven years) is a critical period during the process of child growth, as it occurs at the end of the period of adiposity rebound (between five and seven years) and before the onset of puberty. An early adiposity rebound entails higher adiposity at seven years of age and is a risk factor for obesity in later life [59, 60]. Seven years was therefore a relevant age to study the potential effects of prenatal and childhood exposure on early markers of later obesity. We excluded children who had already initiated puberty from the analysis to consider only childhood adiposity not yet affected by the hormonal modifications of puberty.

The non-monotonic relationship we observed between prenatal chlordecone exposure and adiposity (inverted U-shaped curve) was not entirely unexpected, as it is recognized that EDC compounds may exhibit such patterns [61]. In the Timoun Mother–Child Cohort Study, we previously observed a similar non-monotonic association between prenatal chlordecone exposure and birth weight in overweight and obese mothers [30]. Nonetheless, in the present study, a complementary non-linear analysis (spline regression) did not confirm such a non-linear trend. Consequently, the inverted u-shaped association should be interpreted with caution, and we cannot exclude that it may result from a threshold effect resulting from the categorization of the exposure or a chance finding resulting from residual confounding.

While taking this limitation into account, we also observed that the association between prenatal exposure to chlordecone and adiposity at age seven tended to be stronger among boys. Similarly, sex-dependent effects of perinatal exposure to chlordecone have been frequently reported in animal studies, particularly in the development of behavioral and/or neural function, sexual differentiation, and adult weight gain [29, 62]. These differential effects have been attributed to the estrogenic properties of chlordecone. [26]. In the Timoun Mother–Child Cohort Study, we similarly reported that prenatal exposure to chlordecone was associated with poorer fine motor scores at 18 months of age [33] and lower visual contrast sensitivity at seven years of age, [63] preferentially in boys.

Though non-significant, associations between childhood exposure to chlordecone and adiposity tended to be negative among boys. This observation is consistent with experimental studies conducted in rats, in which early post-natal exposure to chlordecone was associated with significant sex-dependent changes in adult body weight, resulting in lighter males relative to females [29]. In the preceding follow-up at 18 months of age, within in a selected subsample of the initial cohort (*N* = 299), in which children with abnormal birth characteristics (weight, gestational age) or born after pathological pregnancy had been excluded, we showed a significantly elevated BMI to be associated with higher prenatal exposure and a significantly lower BMI with higher early postnatal exposure [31]. Our results at seven years of age suggest a similar pattern, but most of the associations were statistically non-significant. This suggests that the effects observed at earlier ages may be transient and are attenuated with age and exposure to multiple other factors during childhood. It is also possible that adverse medical conditions that had been excluded in the 18 months follow-up (and not at 7 years) may interact with prenatal exposure to chlordecone and adiposity along childhood. However, our mediation analyses of potential intermediate birth outcomes such as birth weight, preterm birth, SGA, and LGA did not show indirect effects of the prenatal chlordecone exposure on adiposity at 7 years.

Mutual adjustment for prenatal and childhood chlordecone concentrations aimed to distengle the effect of exposure during distinct periods of sensitivity. Adjusting for postnatal exposure tended to lower the effect-size of prenatal exposure, particularly in boys. This was partly due to the positive correlation (although moderate or low) between prenatal and postnatal exposures (reflecting common determinants of exposure to chlordecone during pregnancy and childhood) which was higher in boys (rho = 0.15, *p* = 0.08) than in girls (rho = 0.05, *p* = 0.57) (data not shown).

As all cohorts, the Timoun cohort study also suffers from attrition (57% of the initial cohort participated in the follow-up at 7 years). In terms of socio-demographic characteristics, the participating mothers at 7 years were younger and more educated than the non-participants, and mothers from other Caribbean origin, or who smoked during pregnancy were under-represented in the participants (S-Table 6). However no difference was observed between participants and non-participants regarding maternal pre-pregnancy BMI, diabetes and HTA, known as predictors of child adiposity. This suggests that the outcome may not be associated with attrition at 7 years, thus the risk of a Missing Not At Random (MNAR) attrition scenario is limited in our study. Moreover, there was no significant difference in the mean levels of cord blood chlordecone concentrations between participants and non-participants (but the third quartile of exposure was over-represented in the participants). Nevertheless, attrition and missing chlordecone measurements at birth and 7 years contributed to limit the power of some of our analyses, in particular those combining prenatal and childhood exposures (Models 3 in Table 3 and Table 4), after sex stratification and categorization of exposure in quartiles. This may have prevented us from showing significant associations with postnatal exposure, despite trends consistent with those observed at earlier ages.

In our cohort, the prevalence of children reported as exposed to maternal smoking and drinking during pregnancy was too low to study the impact of these factors. The low prevalence of smoking is consistent with the low consumption of tobacco in the FWI population, also in pregnant women, compared to Europe [64–66]. However, drinking during pregnancy may be under-declared in our cohort, despite our efforts to collect it twice, at inclusion (during the third trimester) and after delivery (through an interview on diet during pregnancy). As under-declaration may be differential according to the exposure and/or the outcome levels, residual confounding may be present. We did not consider passive smoking during childhood in our main analyses because it was not correlated with cord blood chlordecone concentrations. A posteriori sensitivity analyses (not shown) confirmed that it did not confound the association between exposure to chlordecone in-utero or in childhood with adiposity at 7 years.

The major strengths of the present study include its prospective design and the multidimensional approach to determining adiposity. The gold standard tool to measure fat accumulation is dual-energy X-ray absorptiometry, but its implementation is complex, invasive, and costly in large epidemiological studies. BMI is widely used as a valuable surrogate measure, but shows limitations in differentiating adipose tissue from lean mass, particularly in the intermediate BMI range [67] and in children and adolescents [68, 69]. The WHtR has been proposed as an efficient alternative that better correlates with visceral abdominal fat and metabolic risk factors in children [70–73]. Skinfold thicknesses (sum of triceps, biceps, subscapular, and suprailiac) have also been studied as adiposity indicators that are more representative of subcutaneous fat than BMI [74, 75]. Most of the epidemiological studies that have investigated obesity and being overweight in children in relation to prenatal exposure to environmental chemical contaminants have been based on weight or BMI only. Only a few included other adiposity indicators, such as waist circumference, the sum of skinfolds, or the waist/height ratio [11, 12]. Here, we chose to use an integrative continuous multidimensional variable that reflects global adiposity based on relevant indicators. The consideration of multiple indicators of adiposity was particularly relevant in our study in the French West Indies, where more than 90% of the population is of African ancestry. The association between BMI and other indicators of adiposity has been shown to differ with ethnicity: black children have less body fat than white children for the equivalent levels of BMI adjusted for age [76]. As a consequence, similar BMI levels may represent different levels of obesity-related risk [77]. Our adiposity score was mainly determined by the BMI z-score, which was modulated by other indicators to reflect individual adiposity more accurately. The definition of this score differed slightly across sexes: the adiposity score correlated more highly with fat mass in girls than boys, whereas no differential factor loading was observed for the other adiposity indicators. Finally, from a statistical perspective, this methodological approach of multiple outcomes through SEM prevented us from replicating the analyses for each adiposity indicator and generating multiple testing.

Another strength of our study is the evaluation of exposure based on measurements of the chlordecone concentrations in blood and the consideration of co-exposure to other organochlorine compounds. The half-life of chlordecone in blood is approximately six months in humans [78]. Thus, a single measurement in cord blood can be considered to be reasonably representative of fetal exposure throughout pregnancy. During childhood, such as at seven years of age, a single determination of plasma chlordecone concentrations reflects the body burden under steady-state conditions and integrates all sources of exposure from various absorption pathways, thus providing a confident estimation of exposure over an extended period. However, variations in chlordecone exposure between birth and seven years of age, during early childhood (breastfeeding, diet diversification), may not have been reflected by the single blood measurement at seven years and the associations observed between exposure and adiposity at seven years of age remain cross-sectional. Moreover, the number of children with exposure measurement below the LOD was not negligible (21.4% for cord blood and 28.3% for childhood measurements), and despite multiple imputation, may have limited our ability to estimate with precision the shape of the association with adiposity at low levels of exposure (within the first quartile of exposure).

In the FWI, the general population is exposed to a mixture of environmental pollutants that may interact with chlordecone exposure or confound the association between chlordecone exposure and child adiposity. But chlordecone contamination of FWI populations is exclusively driven by consumption of local foodstuffs, which in turn are less or not contaminated by widespread pollutants commonly found in Western foodstuffs. In our study, blood concentrations of other POPs that are suspected to promote adiposity in children after prenatal exposure (such as PCB-153 or pp’-DDE) were weakly correlated with chlordecone blood levels. Their integration as co-exposures in sensitivity analyses tended to strengthen the associations, and therefore did not change our conclusions. However, we cannot totally exclude that some residual confounding from other chemical co-exposures exists.

## Conclusions

Our findings in children aged seven years did not allow us to clearly conclude that exposure to chlordecone during critical windows of development contributes to alter weight homeostasis during childhood, before puberty. Growth data collected during further follow-ups of the Timoun Mother–Child Cohort Study will make it possible to search for a possible impact of chlordecone exposure on obesity at puberty.

## Supplementary Information

**Additional file 1.** SUPPLEMENTAL MATERIAL.

## Data Availability

Due to ethical concerns, supporting data cannot be made openly available. The Timoun team can provide the data on request, subject to appropriate approvals. Contact the corresponding author for application.

## Abbreviations

*BMI*: Body Mass Index
*CFI*: Comparative Fit Index
*EDC*: Endocrine-disrupting chemicals
*FWI*: French West Indies
*GAM*: Generalized Additive Model
*GFI*: Goodness-of-Fit statistic
*IQR*: Interquartile Range
*LGA*: Large for Gestational Age
*LOD*: Limit of Detection
*MNAR*: Missing Not At Random
*PCB-153*: Polychlorinated biphenyl, congener 153
*POP*: Persistent Organic Pollutant
*pp’-DDE*: P,p´-dichlorodiphenyldichloroethylene
*RMSEA*: Root Mean Square Error of Approximation
*SEM*: Structural Equation Modeling
*SGA*: Small for Gestational Age
*SRMR*: Standardized Root Mean Square Residual
*WHO*: World Health Organization
*WHtR*: Weight-to-Height Ratio

## References

[1] Abarca-Gómez L, Abdeen ZA, Hamid ZA, Abu-Rmeileh NM, Acosta-Cazares B, Acuin C (2017). Worldwide trends in body-mass index, underweight, overweight, and obesity from 1975 to 2016. Lancet.

[2] Llewellyn A, Simmonds M, Owen CG, Woolacott N (2016). Childhood obesity as a predictor of morbidity in adulthood: a systematic review and meta-analysis. Obes Rev.

[3] Weihrauch-Blüher S, Schwarz P, Klusmann J-H (2019). Childhood obesity: increased risk for cardiometabolic disease and cancer in adulthood ☆. Metabolism.

[4] Grün F, Blumberg B (2006). Environmental obesogens: Organotins and endocrine disruption via nuclear receptor signaling. Endocrinology.

[5] Kelishadi R, Poursafa P, Jamshidi F (2013). Role of environmental chemicals in obesity: a systematic review on the current evidence. J Environ Public Health.

[6] Janesick A, Blumberg B (2011). Endocrine disrupting chemicals and the developmental programming of adipogenesis and obesity. Birth Defects Res Part C - Embryo Today Rev.

[7] Tang-Péronard JL, Heitmann BL, Andersen HR, Steuerwald U, Grandjean P, Weihe P (2014). Association between prenatal polychlorinated biphenyl exposure and obesity development at ages 5 and 7 y: a prospective cohort study of 656 children from the Faroe Islands. Am J Clin Nutr.

[8] Valvi D, Mendez MA, Martinez D, Grimalt JO, Torrent M, Sunyer J (2012). Prenatal concentrations of polychlorinated biphenyls, DDE, and DDT and overweight in children: a prospective birth cohort study. Environ Health Perspect.

[9] Warner M, Aguilar Schall R, Harley KG, Bradman A, Barr D, Eskenazi B (2013). In utero DDT and DDE exposure and obesity status of 7-year-old Mexican-American children in the CHAMACOS cohort. Environ Health Perspect.

[10] Cupul-Uicab LA, Hernández-Ávila M, Terrazas-Medina EA, Pennell ML, Longnecker MP (2010). Prenatal exposure to the major DDT metabolite 1,1-dichloro-2,2-bis(p-chlorophenyl)ethylene (DDE) and growth in boys from Mexico. Environ Res.

[11] Delvaux I, Van Cauwenberghe J, Den Hond E, Schoeters G, Govarts E, Nelen V (2014). Prenatal exposure to environmental contaminants and body composition at age 7–9 years. Environ Res.

[12] Vafeiadi M, Georgiou V, Chalkiadaki G, Rantakokko P, Kiviranta H, Karachaliou M (2015). Association of prenatal exposure to persistent organic pollutants with obesity and cardiometabolic traits in early childhood: the rhea mother–child cohort (Crete, Greece). Environ Health Perspect.

[13] de Cock M, van de Bor M (2014). Obesogenic effects of endocrine disruptors, what do we know from animal and human studies?. Environ Int.

[14] Iszatt N, Stigum H, Verner MA, White RA, Govarts E, Murinova LP (2015). Prenatal and postnatal exposure to persistent organic pollutants and infant growth: a pooled analysis of seven European birth cohorts. Environ Health Perspect.

[15] Tang-Péronard JL, Andersen HR, Jensen TK, Heitmann BL (2011). Endocrine-disrupting chemicals and obesity development in humans: a review. Obes Rev.

[16] Vrijheid M, Casas M, Gascon M, Valvi D, Nieuwenhuijsen M (2016). Environmental pollutants and child health-A review of recent concerns. Int J Hyg Environ Health.

[17] Stratakis N, Rock S, La Merrill MA, Saez M, Robinson O, Fecht D, et al. Prenatal exposure to persistent organic pollutants and childhood obesity: a systematic review and meta-analysis of human studies. Obes Rev. 2022;23. 10.1111/OBR.13383. 10.1111/obr.13383 PMC9512275 34766696

[18] ATSDR. Toxicological Profile for Mirex and Chlordecone. Atlanta: US Department of Health and Human Services; 2020.

[19] Cannon SB, Veazey JM, Jackson RS, Burse VW, Hayes C, Straub WE (1978). Epidemic kepone poisoning in chemical workers. Am J Epidemiol.

[20] Reich MR, Spong JK (1983). Kepone: a chemical disaster in Hopewell. Virginia Int J Heal Serv.

[21] Multigner L, Kadhel P, Rouget F, Blanchet P, Cordier S (2016). Chlordecone exposure and adverse effects in French West Indies populations. Environ Sci Pollut Res.

[22] Cabidoche Y-M, Achard R, Cattan P, Clermont-Dauphin C, Massat F, Sansoulet J (2009). Long-term pollution by chlordecone of tropical volcanic soils in the French West Indies: a simple leaching model accounts for current residue. Environ Pollut.

[23] Dubuisson C, Héraud F, Leblanc J-C, Gallotti S, Flamand C, Blateau A (2007). Impact of subsistence production on the management options to reduce the food exposure of the Martinican population to Chlordecone. Regul Toxicol Pharmacol.

[24] Bocquené G, Franco A (2005). Pesticide contamination of the coastline of Martinique. Mar Pollut Bull.

[25] Guldner L, Multigner L, Héraud F, Monfort C, Pierre Thomé J, Giusti A (2010). Pesticide exposure of pregnant women in Guadeloupe: ability of a food frequency questionnaire to estimate blood concentration of chlordecone. Environ Res.

[26] Hammond B, Katzenellenbogen BS, Krauthammer N, McConnell J (1979). Estrogenic activity of the insecticide chlordecone (Kepone) and interaction with uterine estrogen receptors. Proc Natl Acad Sci U S A.

[27] Eroschenko VP (1981). Estrogenic activity of the insecticide chlordecone in the reproductive tract of birds and mammals. J Toxicol Environ Health.

[28] Kavlock R, Chemoff N, Rogers E, Whitehouse D (1980). Comparative tissue distribution of mirex and chlordecone in fetal and neonatal rats. Pestic Biochem Physiol.

[29] Mactutus CF, Tilson HA (1985). Evaluation of long-term consequences in behavioral and/or neural function following neonatal chlordecone exposure. Teratology.

[30] Hervé D, Costet N, Kadhel P, Rouget F, Monfort C, Thomé J-P, et al. Prenatal exposure to chlordecone, gestational weight gain, and birth weight in a Guadeloupean birth cohort. Environ Res. 2016;151. 10.1016/j.envres.2016.08.004. 10.1016/j.envres.2016.08.004 27560981

[31] Costet N, Pelé F, Comets E, Rouget F, Monfort C, Bodeau-Livinec F, et al. Perinatal exposure to chlordecone and infant growth. Environ Res. 2015;142. 10.1016/j.envres.2015.06.023. 10.1016/j.envres.2015.06.023 26133809

[32] Kadhel P, Monfort C, Costet N, Rouget F, Thome JP, Multigner L (2014). Chlordecone exposure, length of gestation, and risk of preterm birth. Am J Epidemiol.

[33] Boucher O, Simard M-N, Muckle G, Rouget F, Kadhel P, Bataille H (2013). Exposure to an organochlorine pesticide (chlordecone) and development of 18-month-old infants. Neurotoxicology.

[34] Dallaire R, Muckle G, Rouget F, Kadhel P, Bataille H, Guldner L (2012). Cognitive, visual, and motor development of 7-month-old Guadeloupean infants exposed to chlordecone. Environ Res.

[35] Cordier S, Bouquet E, Warembourg C, Massart C, Rouget F, Kadhel P (2015). Perinatal exposure to chlordecone, thyroid hormone status and neurodevelopment in infants: the Timoun cohort study in Guadeloupe (French West Indies). Environ Res.

[36] Glynn AW, Wolk A, Aune M, Atuma S, Zettermark S, Mahle-Schmid M (2000). Serum concentrations of organochlorines in men: a search for markers of exposure. Sci Total Environ.

[37] Multigner L, Ndong JR, Giusti A, Romana M, Delacroix-Maillard H, Cordier S (2010). Chlordecone exposure and risk of prostate cancer. J Clin Oncol.

[38] Debier C, Pomeroy P, Dupont C, Joiris C, Comblin V, Le Boulengé E (2003). Dynamics of PCB transfer from mother to pup during lactation in UK grey seals Halichoerus grypus: differences in PCB profile between compartments of transfer and changes during the lactation period. Mar Ecol Prog Ser.

[39] Bernert JT, Turner WE, Patterson DG, Needham LL (2007). Calculation of serum “total lipid” concentrations for the adjustment of persistent organohalogen toxicant measurements in human samples. Chemosphere.

[40] WHO Multicentre Growth Reference Study Group. 2006. WHO Child Growth Standards: Length/height-for-age, weight-for-age, weight-for-length, weight-for-height and body mass index-for-age: Methods and development. Geneva.

[41] Hu L-T, Bentler PM (2009). Cutoff criteria for fit indexes in covariance structure analysis: conventional criteria versus new alternatives. Struct Equ Model A Multidiscip J.

[42] Kline RB (2011). Principles and Practice of Structural Equation Modeling.

[43] Hooper D, Coughlan J, Mullen MR (2008). Structural equation modelling: Guidelines for determining model fit. Electron J Bus Res Methods.

[44] Helsel DR (2005). Nondetects And Data Analysis: Statistics for censored environmental data.

[45] Jin Y, Hein MJ, Deddens JA, Hines CJ (2011). Analysis of lognormally distributed exposure data with repeated measures and values below the limit of detection using SAS. Ann Occup Hyg.

[46] Voerman E, Santos S, Golab BP, Amiano P, Ballester F, Barros H, et al. Maternal body mass index, gestational weight gain, and the risk of overweight and obesity across childhood: An individual participant data meta-analysis. PLoS Med. 2019;16. 10.1371/journal.pmed.1002744. 10.1371/journal.pmed.1002744 PMC6370184 30742624

[47] Kumar S, Kelly AS (2017). Review of childhood obesity: from epidemiology, etiology, and comorbidities to clinical assessment and treatment. Mayo Clin Proc.

[48] Taveras EM, Gillman MW, Kleinman K, Rich-Edwards JW, Rifas-Shiman SL (2010). Racial/ethnic differences in early-life risk factors for childhood obesity. Pediatrics.

[49] Patro Golab B, Santos S, Voerman E, Lawlor DA, Jaddoe VW, Gaillard R (2018). Influence of maternal obesity on the association between common pregnancy complications and risk of childhood obesity: an individual participant data meta-analysis. Lancet Child Adolesc Heal.

[50] Schisterman EF, Whitcomb BW, Buck Louis GM, Louis T a. Lipid Adjustment in the Analysis of Environmental Contaminants and Human Health Risks. Environ Health Perspect. 2005;113:853–7. 10.1289/ehp.7640. 10.1289/ehp.7640 PMC1257645 16002372

[51] O’Brien KM, Upson K, Cook NR, Weinberg CR (2016). Environmental chemicals in urine and blood: Improving methods for creatinine and lipid adjustment. Environ Health Perspect.

[52] Allison PD (2003). Missing data techniques for structural equation modeling. J Abnorm Psychol.

[53] Allison PD (2012). Handling missing data by maximum likelihood. SAS Glob Forum.

[54] Perenc L, Zajkiewicz K, Drzał-Grabiec J, Majewska J, Cyran-Grzebyk B, Walicka-Cupryś K (2019). Assessment of body adiposity preterm children at the beginning of school age. Sci Rep.

[55] Hong YH, Chung S (2018). Small for gestational age and obesity related comorbidities. Ann Pediatr Endocrinol Metab.

[56] Lu Y, Pearce A, Li L (2020). Weight gain in early years and subsequent body mass index trajectories across birth weight groups: a prospective longitudinal study. Eur J Public Health.

[57] Desquilbet L, Mariotti F (2010). Dose-response analyses using restricted cubic spline functions in public health research. Stat Med.

[58] Rosseel Y (2012). lavaan: an R package for structural equation modeling. J Stat Softw.

[59] Taylor RW, Grant AM, Goulding A, Williams SM (2005). Early adiposity rebound: review of papers linking this to subsequent obesity in children and adults. Curr Opin Clin Nutr Metab Care.

[60] Rolland-Cachera MF, Deheeger M, Maillot M, Bellisle F (2006). Early adiposity rebound: Causes and consequences for obesity in children and adults. Int J Obes.

[61] Toxicology FD (2012). The learning curve. Nature.

[62] Cooper JR, Vodicnik MJ, Gordon JH (1985). Effects of perinatal Kepone exposure on sexual differentiation of the rat brain. Neurotoxicology.

[63] Saint-Amour D, Muckle G, Gagnon-Chauvin A, Rouget F, Monfort C, Michineau L (2020). Visual contrast sensitivity in school-age Guadeloupean children exposed to chlordecone. Neurotoxicology.

[64] Blondel B, Supernant K, du Mazaubrun C, Bréart G (2005). Enquête Nationale Périnatale 2003.

[65] Blondel B, Kermarrec M. Enquête Nationale Périnatale 2010. Paris. 2011. https://solidarites-sante.gouv.fr/IMG/pdf/Les_naissances_en_2010_et_leur_evolution_depuis_2003.pdf.

[66] ARS Guadeloupe (2019). Bulletin de Santé Publique Guadeloupe - TABAC.

[67] Romero-Corral A, Somers VK, Sierra-Johnson J, Thomas RJ, Collazo-Clavell ML, Korinek J (2008). Accuracy of body mass index in diagnosing obesity in the adult general population. Int J Obes.

[68] Javed A, Jumean M, Murad MH, Okorodudu D, Kumar S, Somers VK (2015). Diagnostic performance of body mass index to identify obesity as defined by body adiposity in children and adolescents: a systematic review and meta-analysis. Pediatr Obes.

[69] Jensen NSO, Camargo TFB, Bergamaschi DP (2016). Comparison of methods to measure body fat in 7-to-10-year-old children: a systematic review. Public Health.

[70] Brambilla P, Bedogni G, Heo M, Pietrobelli A (2013). Waist circumference-to-height ratio predicts adiposity better than body mass index in children and adolescents. Int J Obes.

[71] Tuan NT, Wang Y. Adiposity assessments: Agreement between dual-energy X-ray absorptiometry and anthropometric measures in U.S. children. Obesity. 2014;22:1495–504. 10.1002/oby.20689. 10.1002/oby.20689 PMC4037470 24415710

[72] Savva SC, Tornaritis M, Savva ME, Kourides Y, Panagi A, Silikiotou N (2000). Waist circumference and waist-to-height ratio are better predictors of cardiovascular disease risk factors in children than body mass index. Int J Obes.

[73] Kahn HS, Imperatore G, Cheng YJ (2005). A population-based comparison of BMI percentiles and waist-to-height ratio for identifying cardiovascular risk in youth. J Pediatr.

[74] Sardinha LB, Going SB, Teixeira PJ, Lohman TG (1999). Receiver operating characteristic analysis of body mass index, triceps skinfold thickness, and arm girth for obesity screening in children and adolescents. Am J Clin Nutr.

[75] Sarría A, Moreno L, García-Llop L, Fleta J, Morellón M, Bueno M (2001). Body mass index, triceps skinfold and waist circumference in screening for adiposity in male children and adolescents. Acta Paediatr.

[76] Freedman DS, Wang J, Thornton JC, Mei Z, Pierson RN, Dietz WH (2008). Racial/ethnic differences in body fatness among children and adolescents. Obesity.

[77] Wagner DR, Heyward VH (2000). Measures of body composition in blacks and whites: a comparative review. Am J Clin Nutr.

[78] Cohn WJ, Boylan JJ, Blanke R V, Fariss MW, Howell JR, Guzelian PS. Treatment of chlordecone (Kepone) toxicity with cholestyramine. Results Control Clin Trial. 1978;298. 10.1056/NEJM197802022980504. 10.1056/NEJM197802022980504 74014

