# Supplementary material for: Prenatal and childhood exposure to chlordecone and adiposity of seven-year-old children in the Timoun mother–child cohort study in Guadeloupe (French West Indies)
Source: Environ Health. 2022 Apr 19;21:42. doi: 10.1186/s12940-022-00850-2 (PMC9017008; doi:10.1186/s12940-022-00850-2)
Supplement: Supplementary file 1 — Additional file 1. SUPPLEMENTAL MATERIAL. [file 12940_2022_850_MOESM1_ESM.docx]

**Supplemental material**

**Prenatal and childhood exposure to chlordecone and adiposity of seven-year-old children in the Timoun mother-child cohort study in Guadeloupe (French West Indies)**

Costet N, Lafontaine A, Rouget F, Michineau L, Monfort C, Thomé JP, Philippe Kadhel, Multigner L, Cordier S

**Table of Contents**

**S-Figure 1**. Flow chart of the Timoun study (Adiposity at seven years of age)

**S-Table 1**. Associations between covariates included in the models (Chi-square test p-values)

**S-Table 2**. Spearman correlations between adiposity indicators

**S-Table 3**. Factor loadings (unstandardized) of the adiposity latent variable

**S-Figure 2.** Distribution of the adiposity latent variable in boys and girls

**S-Table 4**. Spearman correlations of chlordecone, PCB, and pp’-DDE concentrations

**S-Table 5**. Association between chlordecone concentrations in cord blood and each adiposity indicator at seven years of age (separate multivariate linear regression models)

**S-Figure 3.** General Additive Models (GAM) with restricted cubic splines of the association between chlordecone cord blood concentration and the adiposity score in boys (solid line) and girls (dotted line)

**Results of mediation analyses**

- **S-Figure 4**. Mediation analysis of birth weight as an intermediate outcome for the association between cord blood chlordecone concentrations and adiposity at seven years of age
- **S-Figure 5**. Mediation analysis of preterm birth as an intermediate outcome for the association between cord blood chlordecone concentrations and adiposity at seven years of age
- **S-Figure 6**. Mediation analysis of Small-for-Gestational-Age birth (SGA) as an intermediate outcome for the association between cord blood chlordecone concentrations and adiposity at seven years of age
- **S-Figure 7**. Mediation analysis of Large-for-Gestational-Age birth (LGA) as an intermediate outcome for the association between cord blood chlordecone concentrations and adiposity at seven years of age

S-Table 6. Characteristics of the initial cohort (N=1068) compared to participants (N=592) and non-participants at the follow-up at 7 years.

**S-Figure 1. Flow chart of the Timoun study (Adiposity at seven years of age)**

**
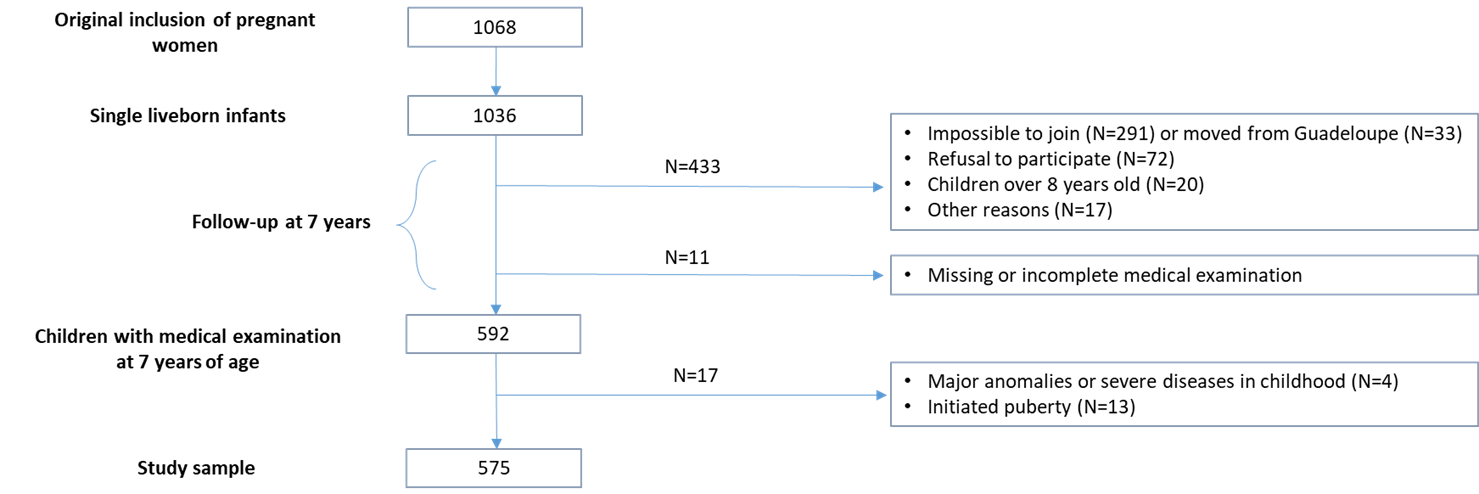
**

**S-Table 1. Associations between covariates included in the models (Chi-square test p-values)**

|  | Maternal BMI | Maternal origin | Education level | Breastfeeding | Obesogenic dietary habits | Exercising | Screen time |
| --- | --- | --- | --- | --- | --- | --- | --- |
| Maternal BMI |  | 0.29 | 0.26 | 0.07 | 0.93 | 0.07 | 0.04 |
| Maternal origin |  |  | <0.0001 | 0.02 | 0.28 | <0.0001 | 0.04 |
| Education level |  |  |  | 0.004 | 0.009 | <0.0001 | 0.39 |
| Breastfeeding |  |  |  |  | 0.28 | 0.04 | 0.83 |
| Obesogenic diet. habits |  |  |  |  |  | 0.01 | 0.1 |
| Exercising |  |  |  |  |  |  | 0.0006 |
| Screen time |  |  |  |  |  |  |  |

| **S-Table 2. Spearman correlations between adiposity indicators** | | | | |  |
| --- | --- | --- | --- | --- | --- |
| **Boys** |  |  |  |  |  |
|  | BMI z-score | % Fat Mass | Sum of skinfolds | WHtR | N |
| BMI z-score | 1 | 0.84 | 0.77 | 0.86 | 282 |
| % Fat Mass |  | 1 | 0.78 | 0.77 | 276 |
| Sum of skinfolds | |  | 1 | 0.70 | 152 |
| WHtR |  |  |  | 1 | 283 |
| **Girls** |  |  |  |  |  |
|  | BMI z-score | % Fat Mass | Sum of skinfolds | WHtR | N |
| BMI z-score | 1 | 0.92 | 0.78 | 0.85 | 291 |
| % Fat Mass |  | 1 | 0.82 | 0.86 | 289 |
| Sum of skinfolds | |  | 1 | 0.76 | 180 |
| WHtR |  |  |  | 1 | 288 |

WHtR: waist-to-height ratio (cm/cm)

Sum of skinfolds: Sum of triceps + subscapular skinfolds (mm)

All *p* < 0.0001

| **S-Table 3. Factor loadings (unstandardized) of the adiposity latent variable** | | | |
| --- | --- | --- | --- |
|  | **Both sexes** | **Boys** | **Girls** |
|  | λ (95%CI) | λ (95%CI) | λ (95%CI) |
| **Raw model^a^** |  |  |  |
| BMI z-score | 1 | 1 | 1 |
| % Fat Mass | 0.10 (0.09; 0.11) | 0.08 (0.08; 0.09) | 0.12 (0.11; 0.12) |
| Sum of skinfolds | 0.11 (0.11; 0.12) | 0.10 (0.09; 0.11) | 0.11 (0.11; 0.12) |
| WtHR | 0.03 (0.03; 0.03) | 0.03 (0.03; 0.03) | 0.04 (0.03; 0.04) |
| **Adjusted model^b^** |  |  |  |
| BMI z-score |  | 1 | 1 |
| % Fat Mass | 0.10 (0.09; 0.11) | 0.08 (0.08; 0.09) | 0.12 (0.11; 0.12) |
| Sum of skinfolds | 0.11 (0.10; 0.12) | 0.10 (0.09; 0.11) | 0.11 (0.10; 0.12) |
| WHtR | 0.03 (0.03; 0.03) | 0.03 (0.03; 0.03) | 0.04 (0.03; 0.04) |

WHtR: waist-to-height ratio (cm/cm)

**^a^**Raw model: adjusted for age and maternal place of birth.

**^b^**Adjusted model: adjusted for exact age at measurement (months), maternal place of birth (Guadeloupe / Martinique, other Caribbean Islands, Europe), maternal BMI before pregnancy (< 18.5, 18.5 to < 25, 25 to < 30, ≥ 30 kg/m²), maternal level of education (< 5, 5 to < 12, ≥ 12 years), duration of breastfeeding (four categories), time spent exercising per week, time spent watching TV or playing videogames per week, obesogenic dietary habits at seven years of age (four categories).

Adjusted models fit indicators:

Both sexes: χ² *p* = 0.00; RMSEA = 0.07 CI_95%_ = [0.06 ; 0.08]; CFI = 0.92; GFI = 1; SRMR = 0.02

Boys: χ² *p* = 0.01; RMSEA = 0.04 CI_95%_ = [0.02; 0.06]; CFI = 0.97; GFI = 1; SRMR = 0.01

Girls: χ² *p* = 0.12; RMSEA = 0.03 CI_95%_ = [0.00; 0.05]; CFI = 0.98; GFI = 1; SRMR = 0.01

**S-Figure 2. Distribution of the adiposity latent variable in boys and girls**


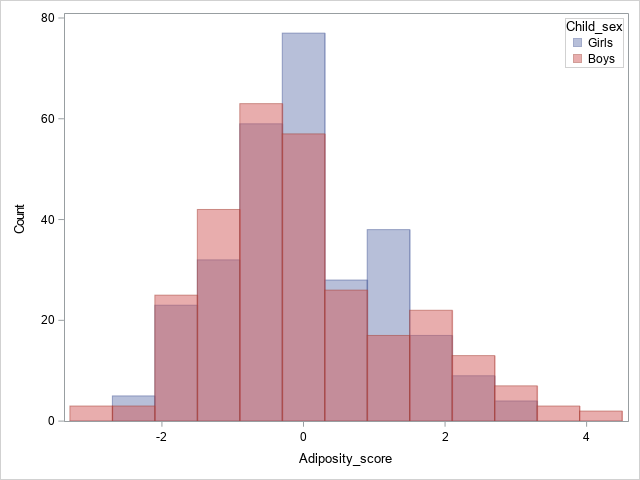


Adiposity is a latent trait defined by four indicators: BMI z-score: WHO references, by sex and age (in months); % Fat mass: estimated by bioelectrical impedance; Sum of skinfolds: sum of the subscapular and tricipital fold measurements; WHtR (Waist-to-Height Ratio): waist circumference (cm) / height (cm).

Sex-specific adiposity scores were constructed, based on the factor loadings presented in S-Table 3 (raw model used here). Scores are expressed in the same unit as the BMI z-score.

**S-Table 4. Spearman correlations of chlordecone, PCB-153, and pp’-DDE concentrations**

|  |  | **In cord blood** | | |  | **At 7 years (blood)** | | |
| --- | --- | --- | --- | --- | --- | --- | --- | --- |
|  |  | Chlordecone | PCB-153 | pp’-DDE |  | Chlordecone | PCB-153 | pp’-DDE |
| **In cord blood** | Chlordecone | 1 | -0.02 | 0.16** |  | 0.09 | -0.12** | 0.02 |
|  | PCB-153 |  | 1 | 0.14** |  | -0.09 | -0.004 | -0.08 |
|  | pp’-DDE |  |  | 1 |  | 0.06 | 0.04 | 0.37*** |
| **At 7 years (blood)** | Chlordecone |  |  |  |  | 1 | -0.11** | 0.004 |
|  | PCB-153 |  |  |  |  |  | 1 | 0.48*** |
|  | pp’-DDE |  |  |  |  |  |  | 1 |

***p* < 0.05, ****p* < 0.0001

S-Table 5. Association between chlordecone concentrations in cord blood and each adiposity indicator at seven years of age (separate multivariate linear regression models)

|  | **BMI z-score** | **Sum of skinfolds (log10)** | **% Fat mass (log10)** | **Waist-to-height ratio** |
| --- | --- | --- | --- | --- |
| **Chlordecone in cord blood (µg/L)** | β ^a^ (95% CI) | β ^a^ (95% CI) | β ^a^ (95% CI) | β ^a^ (95% CI) |
| **Boys** | N=169 | N=149 | N=164 | N=170 |
| < 0.075 | 0 (ref) | 0 (ref) | 0 (ref) | 0 (ref) |
| 0.075 - 0.212 | 0.69 (0.04;1.33) | 0.17 (-0.04;0.37) | 0.10 (-0.05;0.24) | 0.02 (0.00;0.04) |
| 0.212 - 0.382 | 0.72 (0.07;1.36) | 0.25 (0.05;0.44) | 0.14 (-0.01;0.28) | 0.03 (0.01;0.05) |
| ≥ 0.382 | 0.32 (-0.31;0.93) | 0.07 (-0.12;0.26) | -0.01 (-0.15;0.13) | 0.01 (-0.01;0.03) |
| log_10_ | 0.22 (-0.16; 0.60) | 0.08 (-0.04; 0.19) | 0.02 (-0.07; 0.11) | 0.009 (-0.003; 0.020) |
| **Girls** | N=194 | N=176 | N=192 | N=193 |
| < 0.075 | 0 (ref) | 0 (ref) | 0 (ref) | 0 (ref) |
| 0.075 - 0.212 | 0.06 (-0.38;0.49) | 0.00 (-0.15;0.15) | -0.01 (-0.13;0.11) | 0.00 (-0.02;0.02) |
| 0.212 - 0.382 | 0.32 (-0.12;0.76) | 0.11 (-0.05;0.26) | 0.15 (0.02;0.27) | 0.02 (0.00;0.04) |
| ≥ 0.382 | 0.24 (-0.22;0.70) | 0.06 (-0.10;0.21) | 0.09 (-0.04;0.22) | 0.01 (-0.01;0.03) |
| log10 | 0.08 (-0.20; 0.36) | 0.03 (-0.06; 0.12) | 0.05 (-0.03; 0.13) | 0.007 (-0.005; 0.020) |

^a^ Models were adjusted for: exact age at measurement (months) (except for the BMI z-score, which is already corrected for exact age), maternal place of birth (Guadeloupe / Martinique, other Caribbean Islands, Europe), maternal BMI before pregnancy (< 18.5, 18.5 to < 25, 25 to < 30, ≥ 30 kg/m²), maternal level of education (< 5, 5 to < 12, ≥ 12 years), duration of breastfeeding (four categories), time spent exercising per week, time spent watching TV or playing videogames per week, obesogenic dietary habits at seven years of age (four categories), cord blood total lipids (mg/ml, log_10_ scale)

**S-Figure 3. General Additive Models (GAM) (restricted cubic splines) of the association between chlordecone cord blood concentration and the adiposity score in boys (solid line) and girls (dotted line)**


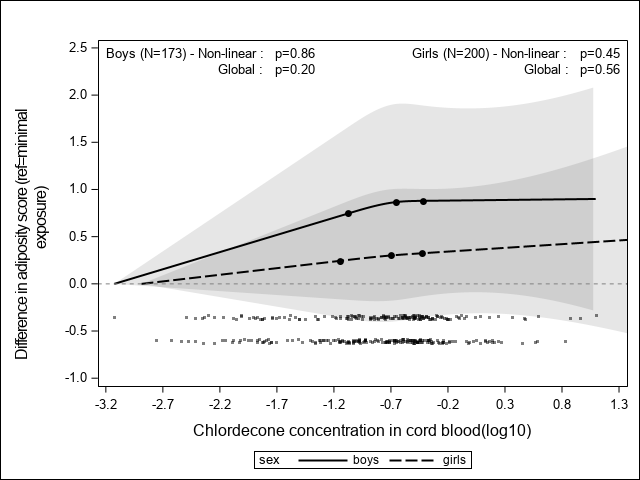


The “adiposity” latent variable was defined as a linear combination of the BMI z-score, the sum of the skinfolds, the WHtR, and the percentage of body fat mass using a Structural Equation Model.

The model was adjusted for: exact age at measurement (months), maternal place of birth (Guadeloupe / Martinique, other Caribbean Islands, Europe), maternal BMI before pregnancy (< 18.5, 18.5 to < 25, 25 to < 30, ≥ 30 kg/m²), maternal level of education (< 5, 5 to < 12, ≥ 12 years), duration of breastfeeding (four categories), time spent exercising per week, time spent watching TV or playing videogames per week, obesogenic dietary habits at seven years of age (four categories), cord blood total lipids (g/L, log_10_ scale).

*Legend* :

X-axis represents chlordecone concentration in cord blood (log-scale)

Y-axis represents the contrast (difference) in the mean adiposity score estimated for each level of exposure, compared to the minimal level of exposure (with its 95% Confidence Interval).

Nodes were placed at the 25^th^, 50^th^ and 75^th^ percentiles.

The significance of an overall association and its non-linear component are tested using Wald Chi2 tests.

**Results of mediation analyses**

Prenatal chlordecone exposure was negatively associated with birth weight, whereas birth weight was positively associated with adiposity at seven years of age for both sexes (S-Figure 4). This resulted in a non-statistically significant indirect effect via birth weight. Prenatal chlordecone exposure was positively associated with preterm birth (S-Figure 5) and SGA (S-Figure 6) for boys only but these birth outcomes were not associated with adiposity. LGA was positively associated with adiposity at seven years of age in girls only but not with prenatal chlordecone exposure (S-Figure 7). Thus, there was no significant indirect effect through intermediate birth outcomes.

All models were adjusted for: exact age at measurement (months), maternal place of birth (Guadeloupe / Martinique, other Caribbean Islands, Europe), maternal BMI before pregnancy (< 18.5, 18.5 to < 25, 25 to < 30, ≥ 30 kg/m²), maternal level of education (< 5, 5 to < 12, ≥ 12 years), duration of breastfeeding (four categories), time spent exercising per week, time spent watching TV or playing videogames per week, obesogenic dietary habits at seven years of age (four categories), cord blood total lipids (g/L, log_10_ scale).

**S-Figure 4. Mediation analysis of birth weight as an intermediate outcome for the association between cord blood chlordecone concentrations and adiposity at seven years of age**


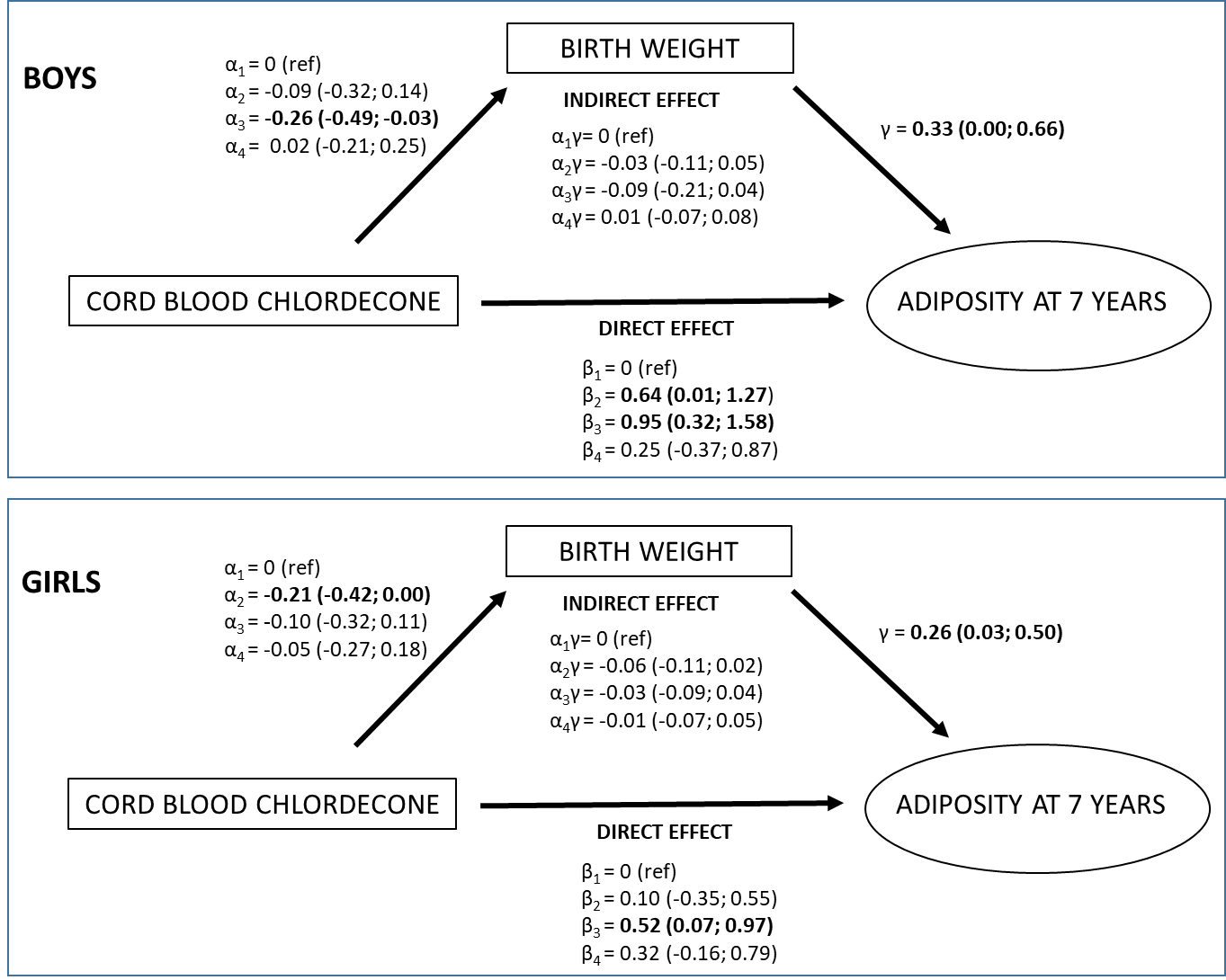


The “adiposity” latent variable was defined from the BMI z-score, the sum of the skinfolds, the WHtR, and the percentage of body fat mass.

α coefficients: adjusted coefficients of cord blood chlordecone concentrations (quartiles) in the regression model predicting “birth weight”.

γ coefficient: adjusted coefficient of “birth weight” in the regression model predicting “adiposity at 7 years”.

β coefficients: adjusted coefficients (including birth weight) of cord blood chlordecone concentrations (quartiles) in the regression model predicting “adiposity at 7 years” (direct effect).

α γ coefficient: indirect effect of cord blood chlordecone concentrations on “adiposity at 7 years” through “birth weight”.

**S-Figure 5. Mediation analysis of preterm birth as an intermediate outcome for the association between cord blood chlordecone concentrations and adiposity at seven years of age**


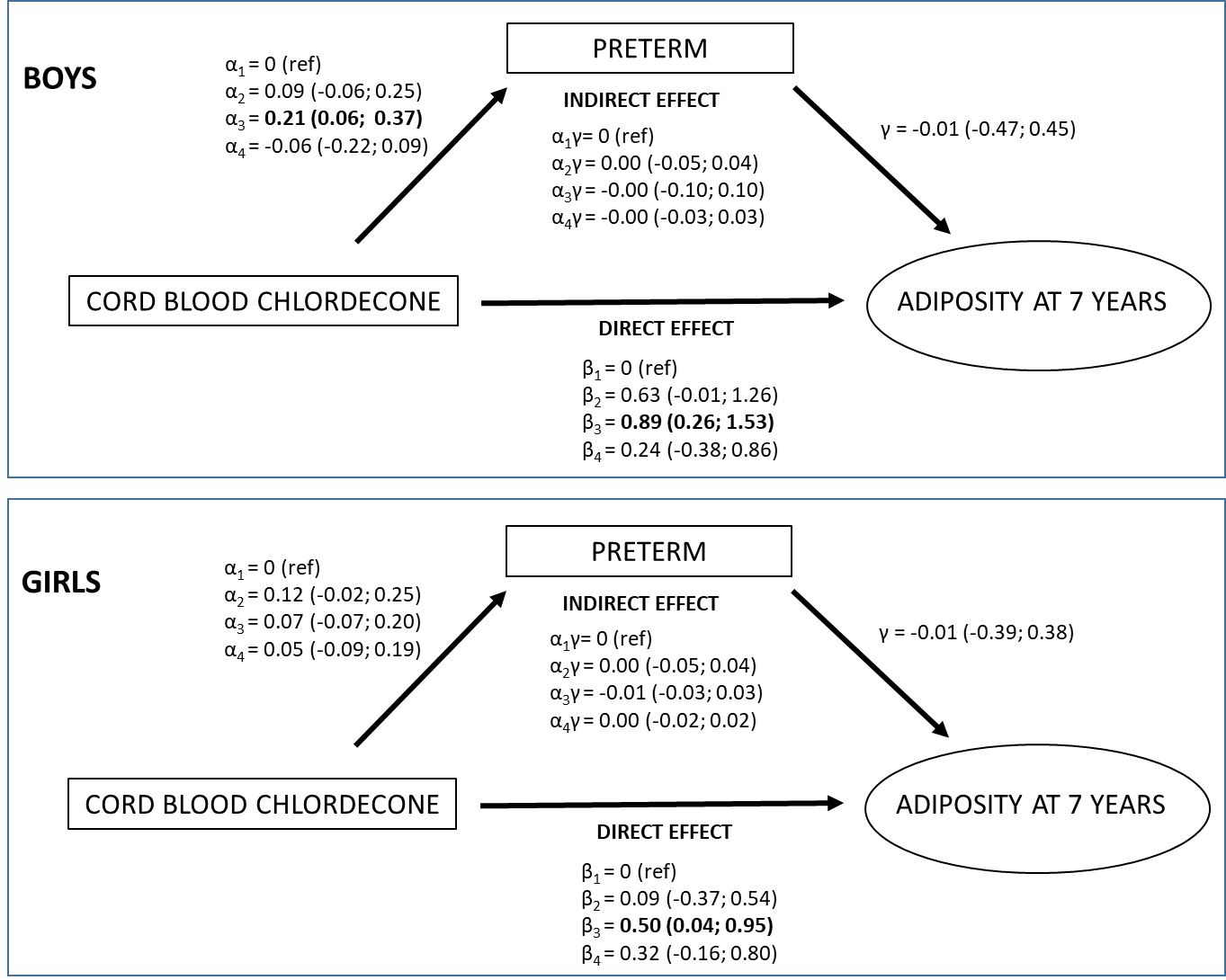


The “adiposity” latent variable was defined from the BMI z-score, the sum of the skinfolds, the WHtR, and the percentage of body fat mass.

α coefficients: adjusted coefficients of cord blood chlordecone concentrations (quartiles) in the regression model predicting “preterm birth”.

γ coefficient: adjusted coefficient of “preterm birth” in the regression model predicting “adiposity at 7 years”.

β coefficients: adjusted coefficients (including preterm birth) of cord blood chlordecone concentrations (quartiles) in the regression model predicting “adiposity at 7 years” (direct effect).

α γ coefficient: indirect effect of cord blood chlordecone concentrations on “adiposity at 7 years” through “preterm birth”.

**S-Figure 6. Mediation analysis of Small-for-Gestational Age birth (SGA) as an intermediate outcome for the association between cord blood chlordecone concentrations and adiposity at seven years of age**

**
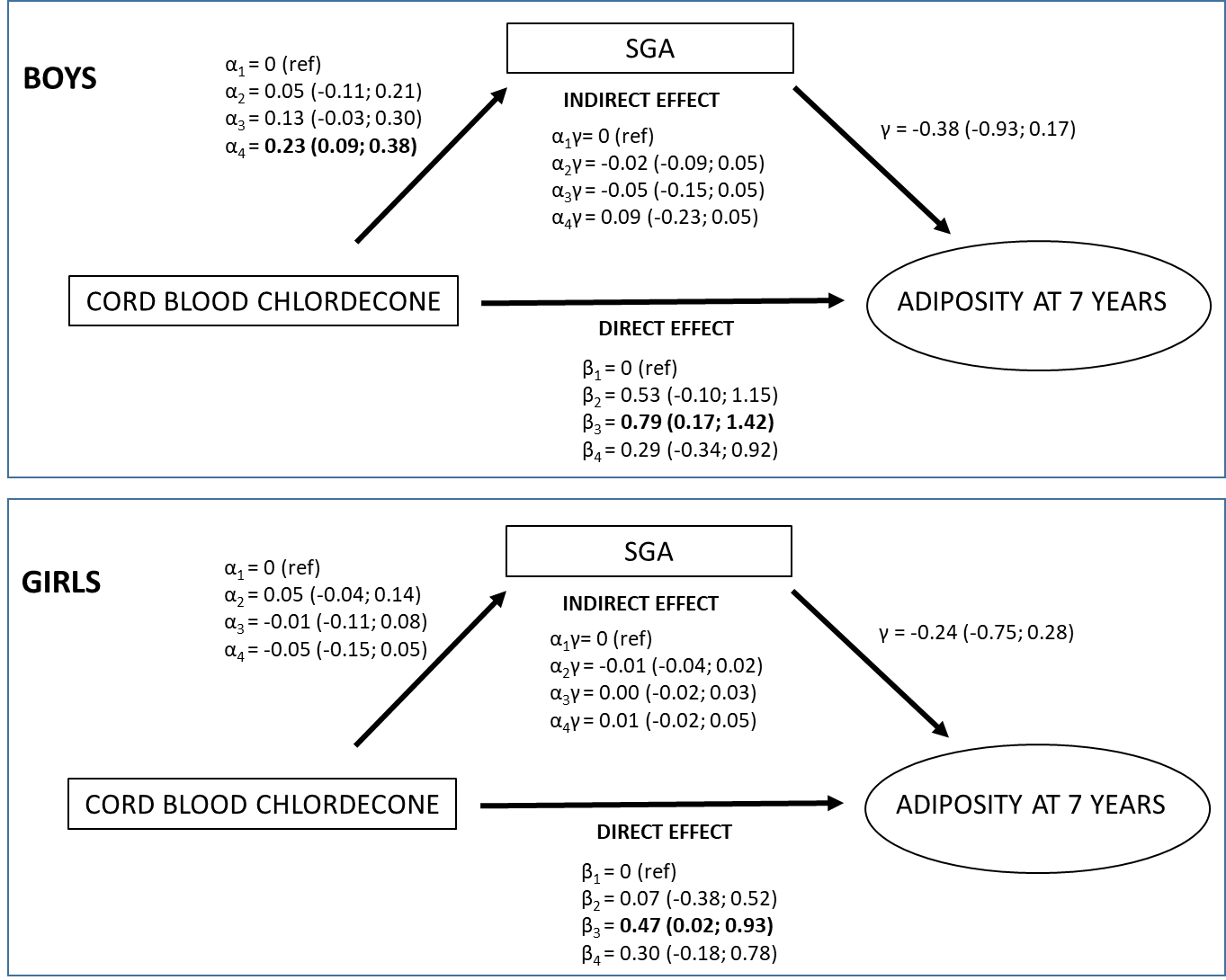
**

The “adiposity” latent variable was defined from the BMI z-score, the sum of the skinfolds, the WHtR, and the percentage of body fat mass.

α coefficients: adjusted coefficients of cord blood chlordecone concentrations (quartiles) in the regression model predicting “SGA”.

γ coefficient: adjusted coefficient of “SGA” in the regression model predicting “adiposity at 7 years”.

β coefficients: adjusted coefficients (including SGA) of cord blood chlordecone concentrations (quartiles) in the regression model predicting “adiposity at 7 years” (direct effect).

α γ coefficient: indirect effect of cord blood chlordecone concentrations on “adiposity at 7 years” through “SGA”.

**S-Figure 7. Mediation analysis of Large-for-Gestational Age birth (LGA) as an intermediate outcome for the association between cord blood chlordecone concentrations and adiposity at seven years of age**

**
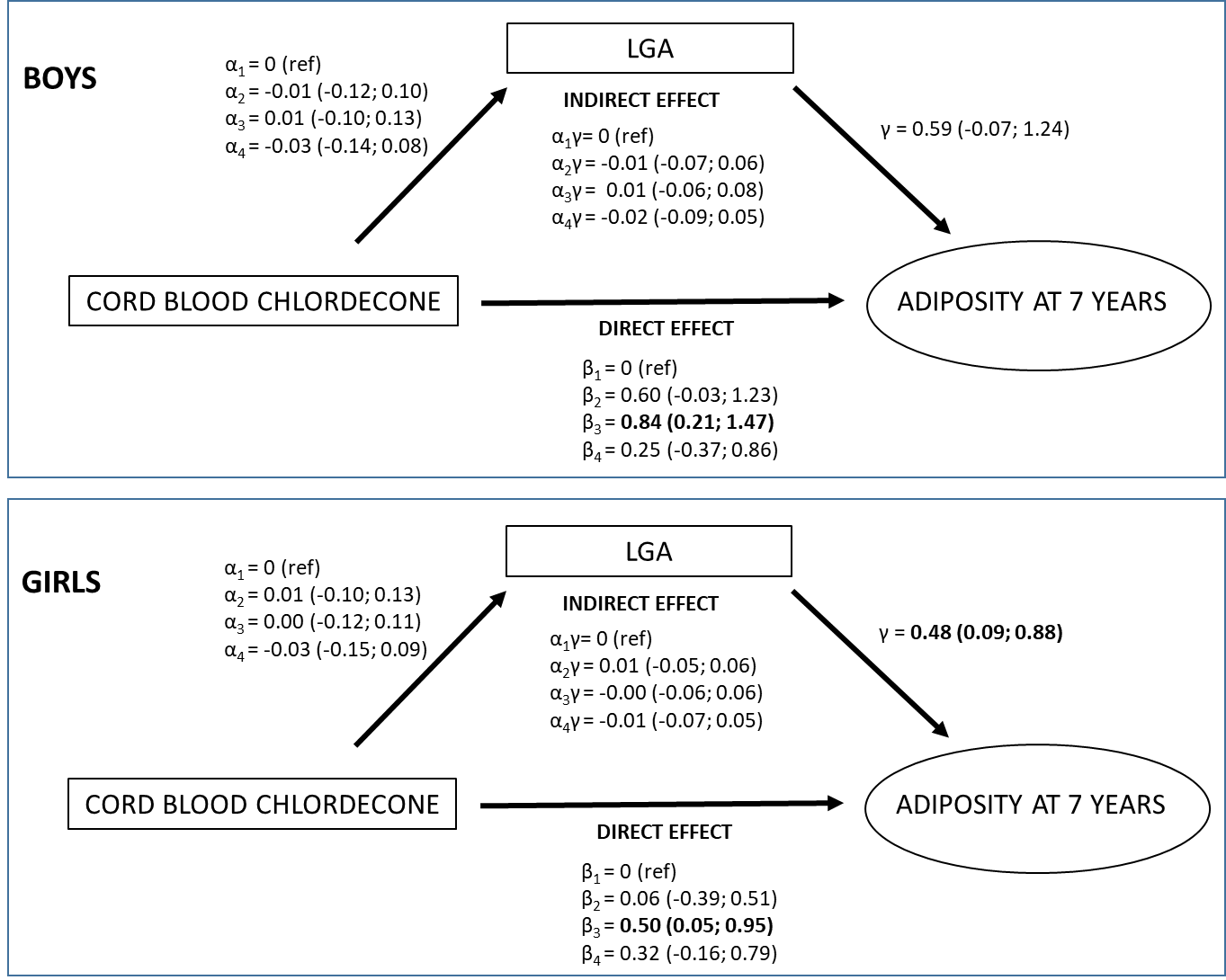
**

The “adiposity” latent variable was defined from the BMI z-score, the sum of the skinfolds, the WHtR, and the percentage of body fat mass.

α coefficients: adjusted coefficients of cord blood chlordecone concentrations (quartiles) in the regression model predicting “LGA”.

γ coefficient: adjusted coefficient of “LGA” in the regression model predicting “adiposity at 7 years”.

β coefficients: adjusted coefficients (including LGA) of cord blood chlordecone concentrations (quartiles) in the regression model predicting “adiposity at 7 years” (direct effect).

α γ coefficient: indirect effect of cord blood chlordecone concentrations on “adiposity at 7 years” through “LGA”.

| **S-Table 6. Characteristics of the initial cohort (N=1068) compared to participants (N=592) and non-participants at the follow-up at 7 years** | | | | | | | |
| --- | --- | --- | --- | --- | --- | --- | --- |
|  | **All (N=1068)** | | **Non-participants (N=476)** | | **Participants (N=592)** | | **p-value** ^a^ |
|  | N | % or  mean (std) | N | % or  mean (std) | N | % or mean (std) |  |
| **Pregnancy and birth** |  |  |  |  |  |  |  |
| Maternal age | 1068 | 31.7 (6.6) | 476 | 30.1 (7.2) | 592 | 31.9 (6.6) | <0.0001 |
| Maternal place of birth |  |  |  |  |  |  | 0.05 |
| French West Indies | 835 | 78.2 | 357 | 75.0 | 478 | 80.7 |  |
| Other Caribbean Islands | 119 | 11.1 | 64 | 13.4 | 55 | 9.3 |  |
| Europe | 114 | 10.7 | 55 | 11.6 | 59 | 10.0 |  |
| Maternal education (yrs) |  |  |  |  |  |  | 0.04 |
| < 5 | 64 | 6.0 | 36 | 7.6 | 28 | 4.7 |  |
| 5–12 | 751 | 70.3 | 340 | 71.4 | 411 | 69.4 |  |
| > 12 | 253 | 23.7 | 100 | 21.0 | 153 | 25.8 |  |
| Maternal BMI |  |  |  |  |  |  | 0.32 |
| Underweight | 67 | 6.4 | 34 | 7.3 | 33 | 5.7 |  |
| Normal | 554 | 53.0 | 248 | 53.4 | 306 | 52.6 |  |
| Overweight | 229 | 21.9 | 91 | 19.6 | 138 | 23.7 |  |
| Obese | 196 | 18.7 | 91 | 19.6 | 105 | 18.0 |  |
| Parity |  |  |  |  |  |  |  |
| Null | 392 | 36.7 | 188 | 39.5 | 204 | 34.5 | 0.005 |
| 1 | 312 | 29.2 | 115 | 24.2 | 197 | 33.3 |  |
| 2 and above | 364 | 34.1 | 173 | 36.3 | 191 | 32.3 |  |
| Tobacco during pregnancy | 57 | 5.3 | 39 | 8.2 | 18 | 3.0 | 0.0002 |
| Alcohol during pregnancy | 28 | 2.8 | 16 | 3.6 | 12 | 2.1 | 0.17 |
| Hypertension | 130 | 12.6 | 58 | 12.6 | 72 | 12.6 | 0.99 |
| Diabetes | 146 | 14.2 | 66 | 14.3 | 80 | 14.0 | 0.90 |
| Preterm birth | 175 | 16.4 | 88 | 18.5 | 87 | 14.7 | 0.10 |
| Small for gestational age | 110 | 10.3 | 58 | 12.2 | 52 | 8.8 | 0.07 |
|  |  |  |  |  |  |  |  |
| **Exposure to organochlorines** |  |  |  |  |  |  |  |
| **Chlordecone in cord blood** | 688 |  | 305 |  | 383 |  | 0.05 |
| < 0.075 | 185 | 26.9 | 89 | 29.2 | 96 | 25.1 |  |
| 0.075 - 0.212 | 178 | 25.9 | 82 | 26.9 | 96 | 25.1 |  |
| 0.212 - 0.382 | 146 | 21.2 | 50 | 16.4 | 96 | 25.1 |  |
| ≥ 0.382 | 179 | 26.0 | 84 | 27.5 | 95 | 24.8 |  |
| **PCB153 in cord blood (µg/L)** | 689 |  | 307 |  | 382 |  | 0.14 |
| 0 | 213 | 30.9 | 97 | 31.6 | 116 | 30.4 |  |
| 0 - 0.062 | 155 | 22.5 | 80 | 26.1 | 75 | 19.6 |  |
| 0.062 - 0.144 | 162 | 23.5 | 67 | 21.8 | 95 | 24.9 |  |
| ≥ 0.144 | 159 | 23.1 | 63 | 20.5 | 96 | 25.1 |  |
| **pp’-DDE in cord blood (µg/L)** | 689 |  | 307 |  | 382 |  | 0.07 |
| < 0.096 | 190 | 27.6 | 95 | 30.9 | 95 | 24.9 |  |
| 0.096 - 0.280 | 184 | 26.7 | 88 | 28.7 | 96 | 25.1 |  |
| 0.280 - 0.684 | 163 | 23.7 | 67 | 21.8 | 96 | 25.1 |  |
| ≥ 0.684 | 152 | 22.0 | 57 | 18.6 | 95 | 24.9 |  |

^a^ Chi-square Tests (qualitative characteristics) and t-tests (continuous characteristics) to compare participating *vs* non-participating women.
